# Unresolved ethical questions of mHealth apps for Alzheimer’s disease prevention

**DOI:** 10.1007/s11019-025-10272-9

**Published:** 2025-05-26

**Authors:** Karina Korecky, Silke Schicktanz

**Affiliations:** Department of Medical Ethics and History of Medicine, University Medical Center Göttingen, Göttingen, Germany

**Keywords:** Ethics of dementia prevention, mHealth apps, Responsibilization, Data protection, Stigma

## Abstract

In recent years, medical research has sparked hope that up to a third of dementia cases could be prevented. This optimism is driven by a shift in the understanding of dementia and, in particular, Alzheimer’s Disease (AD)—from being a rapid-onset brain disease in later life to a condition strongly linked to lifestyle factors, progressing slowly and gradually through asymptomatic, pre-symptomatic, and symptomatic stages with varying degrees of severity. Accompanying this evolving perception, the use of mobile healthcare applications (mHealth apps) based on dementia prevention research has been on the rise. Health policymakers and companies increasingly advocate for these apps. However, concerns remain about the medical quality of such mHealth apps for dementia prevention. Bioethical research has highlighted significant challenges associated with their use. This paper critically examines dementia prevention strategies through the lenses of mHealth technologies. Exploring four mHealth apps for dementia prevention as case studies, we identify and analyze unsolved ethical issues related to primary, secondary, and tertiary prevention. Hereby we offer a new perspective on familiar ethical dilemmas in dementia prevention, and emphasize the need to examine potentially intensified challenges in the context of digital health in the future in more depth.

## Background: The “new dementia” and its ethical implications

The growing public focus on dementia prevention arises from two key scientific developments: the persistent failure of pharmaceutical research to discover a cure despite nearly three decades of substantial investment, and a fundamental shift in how dementia and, in particular, Alzheimer’s Disease (AD) are conceptualized. Whether the new treatment of early AD patients with the monoclonal antibody Lecanemab for early AD patients will be the radical game change, is not yet clear (McDade et al. 2022). Once regarded as brain diseases with rapid onset in later life, these conditions are now understood as progressively developing over a long period, with varying degrees of severity. Their symptomatic states can often be predicted well in advance, not only through psychological and cognitive tests but also via biochemical “biomarkers” (Livingston et al. 2020, p. 414) detectable through laboratory testing. This gradual development of dementia and its predictability have fueled optimism that prevention might be possible. A highly cited first report in *The Lancet* suggested that up to a third of dementia cases could be prevented by addressing modifiable risk factors (Livingston et al. 2017). An updated report (Livingston et al. 2020) identified specific risk factors, including low education levels, hearing loss, traumatic brain injury, hypertension, excessive alcohol consumption (over 213 ml per week), obesity, smoking, depression, social isolation, physical inactivity, air pollution, and diabetes. Highlighting the continuum of dementia’s development across the life course, the report emphasizes that these risk factors span over different life stages (Livingston et al. 2020, p. 414). Epidemiological data further reinforces this view, associating reductions in dementia incidence in Western countries with improvements in education, healthcare, and social welfare systems (Wu et al. 2017). This reframing repositions dementia as a condition tied closely to lifestyle factors and no longer confined to the final stage of life, often referred to as the “fourth” age (Higgs and Gilleard 2017).

This reconceptualization has significant implications. It prompts a health policy shift not only from diagnosis to prediction (Leibing and Schicktanz 2021), but also from the pursuit of a cure in the fourth life stage to the promotion of primary, secondary, and tertiary prevention. Primary prevention focuses on avoiding or delaying the onset of dementia. Secondary prevention involves early detection to enable timely treatment and to slow down the disease progression. Tertiary prevention encompasses measures aimed at preserving residual functions, rehabilitation, and maintaining quality of life after the disease has advanced.

Prevention strategies operate on two levels: individual behavior and lifestyle changes, and broader health policy and collective efforts to reduce risk factors (e.g. by reducing social exposure to risk factors or increasing resilience). However, the public discourse and mHealth technologies often place the primary responsibility for dementia prevention on individuals (Petersen and Schicktanz 2021), even though current research does not provide conclusive evidence regarding the extent to which lifestyle choices influence dementia onset (see overviews by Lawless et al. 2018; Leanza 2021). The prevailing assumption appears to be that dementia prevention efforts, at any level, are inherently beneficial and thus preferable to inaction. This aligns with the broader moral premise that promoting a healthy lifestyle causes no harm, particularly in the absence of curative treatments (Schicktanz 2021). Nevertheless, there is no guarantee that any of the currently recommended preventive measures will effectively prevent AD. Moreover, AD prevention interventions and technologies are shaped by structural and institutional inequalities throughout the life course, e.g. such as socio-economical differences in access to education, health information, and affordability of healthy food.

In recent years, several studies have addressed the potential for individualized dementia prevention strategies to exacerbate existing inequalities (Daly 2023, 2024; Li et al. 2023; Wilson and Anstey 2023; Jones et al. 2024). Drawing on debates in medical sociology about *responsibilization*—the process of making individuals accountable for their own health (e.g., Petersen and Lupton 1996; Rier 2022)—bioethical scholarship has highlighted the limitations of individual health-related responsibilities in the context of AD prevention, (Schicktanz and Schweda 2012; Schweda and Pfaller 2021). These scholars argue that individual responsibility for health outcomes can only be justifiably attribute when: (a) the individual is aware that a specific action that will lead evidently to a particular outcome, (b) the individual intentionally performs the action, and (c) the action causally influences the expected outcome. Based on this framework, assigning responsibility for AD prevention solely to individuals is unjustifiable. Individuals lack personal causal influence over several critical risk factors for dementia, such as low education or air pollution.

In the context of mHealth apps—more generally observed—, the trend toward responsibilization appears to be intensifying (De Proost and Segers 2024; Meier et al. 2024). Mobile healthcare technologies, it is worried, can bypass the traditional mediation of healthcare professionals, not only place additional responsibilities on patients—such as tracking health data, tasks previously shared with or managed by professionals (Lucivero and Jongsma 2018, p. 687). However, there is also the claim to “empower” individuals by enhancing their autonomy and, in turn, their accountability for their health. Research has critically examined the concept of empowerment within the digital health and dementia assistive technology debate (Löbe and AboJabel 2022). Recent critiques challenge the digital health industry’s often made claims about empowering mHealth users, highlighting that such promises may be misleading. As one study observes, “instead of empowerment, it is plausible that m-health technology merely facilitates a feeling of empowerment” (Kreitmair 2024, p. 488). Additionally, scholars have noted logical inconsistencies and overly narrow definitions of empowerment in the context of mHealth. For instance, Gray et al. (2024) argue that “these digital health technologies encourage users to adopt an individualized conception of autonomy, one that may weaken the doctor–patient relationship, undermine shared decision-making practices, and ultimately fail to improve health outcomes for those who need it the most” (Gray et al. 2024, p. 1). Given the growing role of mHealth apps in dementia prevention, we stress the importance of context-sensitive ethical assessments tailored to specific domains and conditions.

## A qualitative approach to the study of apps

The interest in dementia prevention and related apps is steadily growing (Butler et al. 2018), with some apps already integrated into public health services. Dementia prevention apps are utilized across all three prevention phases. In primary prevention, smartphone apps are promoted to manage training programs aimed at reducing dementia risk (Bonnechère and Sahakian 2020). In secondary prevention, apps are employed to assess whether an individual may be developing dementia (Astell et al. 2019; Kapeller and de Boer 2024). For tertiary prevention, apps provide safe online platforms for people with dementia to communicate and engage with others (Elfaki and Alotaibi 2018).

Despite their popularity, relatively few psychological and medical studies have rigorously evaluated the medical value of mHealth apps in general (Terhorst et al. 2020; Lagan et al. 2021) or apps specifically claiming to enhance brain fitness and support dementia prevention (Strobach and Huestegge 2017; Zeiler et al. 2023). Zeiler et al. (2023) conclude that most apps targeting individuals with Mild Cognitive Impairment (MCI) lack robust evidence and cannot be scientifically validated. These findings align with calls from other researchers for the establishment of reliable, independent, and international quality certifications to safeguard consumers and patients in the increasingly saturated mHealth app market (Essén et al. 2022).

In this paper, we propose that psychological, neuro-scientific, and health science research on dementia prevention apps should be complemented by examinations of the ethical dimensions of these applications. A closer look at available apps (in English or German) reveals a wide range, from cognitive fitness and self-testing apps to self-tracking apps and those promising rehabilitation and maintenance of brain function. We selected four apps from the market, each representing a different type of app, and conducted a systematic analysis using the qualitative *walkthrough method for the study of apps* (Light et al. 2018; Meister and Slunecko 2021; Duguay and Gold-Apel 2023). This method integrates approaches from science and technology studies (specifically Latour’s Actor-Network Theory) with cultural studies techniques and is one of the few qualitative methods for studying apps: “The walkthrough method is a way of engaging directly with an app’s interface to examine its technological mechanisms and embedded cultural references to understand how it guides users and shapes their experiences” (Light et al. 2018, p. 882).

The walkthrough method provides a controlled framework for interacting with an app to identify key technological mechanisms and cultural meanings. It collects data on the app’s “vision” (purpose, target user base, and use scenarios), “operating model” (business strategy and revenue sources), “governance” (regulation of user activities), user interface design, functions and features, textual content and tone, symbolic representation, and options for suspending, closing, or deleting user data (Light et al. 2018). For ethical assessment, we added three dimensions–”data protection”, “informed consent”, and analyzed the “concepts and strategies of prevention” as conveyed through app usage. The steps of the walkthrough follow the chronology of a typical app experience, including onboarding, everyday use, and offboarding, with the researcher taking field notes in a research journal. Due to geographic restrictions, only two of the four apps could be fully analyzed through a walkthrough (*memodio*, *Lumosity*,* see below*), while information on the other two apps (*BrainTest*, *SingFit*,* see below*) was gathered from company websites and additional sources (e.g., *YouTube*, newspaper and magazine articles, app store reviews). In the case of *BrainTest*, the paper-and-pen test on which the app is based, was also accessible for comparison.

## Dementia prevention apps: Four case studies

The German start-up *memodio*, founded by the University of Potsdam, has developed an app offering brain training, exercises, and dietary guidance for individuals with MCI or early-stage dementia. Launched in 2021, the app is certified as a medical device and is eligible for reimbursement through three German statutory health insurance providers with a doctor’s prescription. However, since the app focuses on prevention rather than treatment for a specific medical condition, it is not classified as a digital health application (DiGA). To date, German authorities have approved only one app for early-stage dementia patients as a DiGA: *NeuroNation MED*, developed by the Berlin-based company Synaptikon.^1^ Another start-up, *Mindahead*, plans to seek certification for its product in 2025.^2^ The market for mHealth apps in Germany has grown significantly since the Digital Healthcare Act of 2019, which introduced fast-track approvals and reimbursement opportunities through statutory health insurance providers. Manufacturers of DiGAs are required to demonstrate “positive health care effects”^3^ to qualify. Although not certified as a DiGa, *memodio* positions itself as a medical intervention and describes its primary objective as “slowing down the progression of dementia.” 4

The app *Lumosity*, developed by California-based Lumos Labs, was launched in 2007 and has become one of the most prominent brain fitness apps, with over 100 million downloads as of 2022, according to its developer.^5^ The company received significant attention in 2016 when it settled charges brought by the Federal Trade Commission (FTC). The FTC accused Lumos Labs of deceptive advertising, alleging that it marketed *Lumosity* under the false claim of scientific evidence, exploiting consumer fears about age-related cognitive decline. The app was framed as “brain training” capable of staving off memory loss, dementia, and even Alzheimer’s disease, claims the FTC deemed misleading.^6^ This case highlighted widespread criticism of brain fitness apps among neuroscientists and spurred further research into their effectiveness (Kable et al. 2017). In response, Lumos Labs established its own research lab and has since published studies supporting the app’s positive effects. *Lumosity* offers a variety of games, puzzles, riddles, and reaction-based activities designed to train skills such as memory, processing speed, and problem-solving. Unlike *memodio*, *Lumosity* does not claim to provide medical services and is not approved by the U.S. Food and Drug Administration (FDA) as a digital health service. By contrast, the FDA has approved other mental health apps, such as *EndeavorRx*, a game-based app for children diagnosed with ADHD. Despite lacking FDA approval, *Lumosity* emphasizes the scientific foundation of its games, which are based on cognitive science principles. Both *memodio* and *Lumosity* fall into the category of apps for primary prevention, as they do not require or provide a medical diagnosis of disease or dysfunction.

The *BrainTest* app, launched in 2017 by a company now based in the Cayman Islands, straddles the line between primary and secondary prevention. On one hand, it positions itself as a tool for the early detection of dementia, offering anyone over the age of 13 who suspects cognitive impairments a quick at-home test with results available within a few days. In this sense, the app aligns with the principles of secondary prevention, which focuses on identifying and addressing early stages of dementia. On the other hand, the app’s terms of service explicitly state that it is not a diagnostic tool: “We do not provide any medical or clinical services and do not diagnose, treat, or manage any illness, disease, or condition. In particular, the Service is not a diagnostic tool or device.”^7^ Instead, it serves as a decision-support tool to determine whether consulting a doctor might be necessary. This places the app within the domain of primary prevention. *BrainTest* is based on a paper-and-pen tool for dementia testing, which can be self-administered or facilitated by a primary care physician. It retains features typical of such tests, requiring use on a tablet rather than a phone or desktop computer, as drawing—rather than just typing or tapping—is one of the abilities assessed. The first test establishes a baseline for future comparisons. Test results are accompanied by a brief explanatory video from a physician, who has no additional information about the user beyond their test score.

The fourth app in our analysis falls into the category of tertiary prevention and, unlike the others, is designed not for patients or concerned users but for caregivers and therapists. *SingFit*, developed by a California-based start-up, was launched in 2011 and provides tools for “therapeutic” music sessions in assisted living communities and other caregiving contexts. While it can be used for solo singing, promotional videos and customer feedback suggest it is primarily employed in group settings: “We empower healthcare professionals and individuals to utilize music as medicine.”^8^ Like the other apps, *SingFit* emphasizes its scientific foundation and therapeutic value. However, it stops short of claiming to be a medical device (unlike *memodio*): “*SingFit* is therapeutic music; it is not music therapy.” 9 The app offers features such as a personalized music library, guided lyrics (where lyrics are spoken immediately before being sung to assist participants), and options for recording songs. Additionally, caregivers and session organizers are provided with conversation prompts, instructional videos on app usage in group settings, and strategies for designing effective sessions.

## Ethical issues of primary prevention apps

A major advantage of mHealth apps for primary dementia prevention is that they promise healthcare anytime and anywhere, overcoming geographic, temporal, and organizational barriers (Hafdi et al. 2021). In low- and middle-income countries, where mobile phone rates are high, technological advances can help address some of the most pressing health inequalities. However, it has been reported that people living in environments not conducive to health-conscious lifestyles generally do not tend to use these apps (Forlini and Hall 2017). Moreover, it seems that the more an app aims to provide therapeutic dementia prevention, the more it considers the social context of its users. For example, the games offered by *Lumosity* can be played anywhere, regardless of the healthcare systems or medical support available. However, *memodio* makes it clear on its welcome screen that its use does not replace medical advice from a doctor and, although it was not addressed during the walkthrough, it is possible that the app may at some point advise users to consult with a healthcare professional. Similarly, *BrainTest*, which cannot be downloaded everywhere due to geo-blocking, also refers users to healthcare providers. Both apps clearly do not replace professional healthcare, but rather serve as supplements to existing systems.

The second ethical issue, already raised in the charges against *Lumosity*, will become more significant in the coming years as health insurances increasingly reimburse mHealth applications: Do these apps improve long-term cognitive health? So far, there is no conclusive scientific evidence showing a systematic influence on risk factors (Butler et al. 2018; Schürmann and Trachsel 2023) or the effectiveness of dementia prevention apps (Kaplan and Ranchordás 2019). In the case of brain fitness apps, meta-studies have not found improvements beyond enhancing users’ ability to operate the app itself (Stojanoski et al. 2021). The key question here is whether apps like *Lumosity* or *memodio* facilitate the transfer of skills learned in the app to real-world situations and hence improve everyday-life cognition.

The third ethical issue relates to the indirect effects of primary prevention apps, whether they lean toward entertainment, such as *Lumosity* (represented by its orange-colored icon, symbolizing fun and creative activities while simultaneously stimulating the brain), position themselves as medical tools, like *memodio* (featuring a stylized human brain outlined in purple—the color purple is associated with Alzheimer’s awareness), or occupy a middle ground between fun and medicine, like *BrainTest* (whose icon features an arch transitioning from purple to orange). These apps suggest that brain fitness or therapy for MCI primarily involves solving algebra problems and memorizing words and images. They share the most common approach in cognitive stimulation apps, which is the recall of images, words or symbols (Ye et al. 2023) (*memodio* does integrate also motivations for physical exercise). All medical evidence relating dementia and AD to biographical memory, emotional and planning skills, personal identity and a changing relation to the body, do not seem to be addressed. Also rarely are addressed psychological reactions to the realization of memory loss like attempts to cope, or emotional reactions like shame and anxiety (e.g. *memodio* does ask about “being scared to get dementia” in the onboarding process). The standardized and individual nature of interaction with these apps conveys a reductive view of dementia—primarily as difficulties with memorization and recall, akin to a malfunctioning input-output system of knowledge. This framing emphasizes personal effort and adherence to the app’s requirements; *memodio*, for instance, recommends daily use for 25–30 min at the same time each day and sends push notifications to encourage compliance. However, dementia prevention studies have consistently shown that lifestyle changes, such as those promoted by apps, have only a limited impact on dementia prevalence compared to broader structural and institutional factors. For instance, access to environments conducive to physical activity, healthy food, and educational opportunities remains crucial. Individual lifestyles are deeply shaped by socioeconomic contexts and cannot be regarded as solely personal choices (Forlini and Hall 2017). Once established, these lifestyles are difficult to change without substantial external support (Whitehouse 2019). Public health efforts, such as improving health literacy and creating supportive environments, are essential to enable meaningful lifestyle changes. Approaches focused on individual responsibility alone disproportionately benefit those with the socioeconomic means to adopt a brain-healthy lifestyle. Without accompanying collective efforts, such strategies risk exacerbating existing health inequalities (Forlini and Hall 2017). Dementia prevention campaigns centered on personal lifestyles, without addressing systemic barriers, may even lead to competition for public resources, favoring those who already have access and further widening health disparities (Walsh et al. 2022). Consequently, population-wide approaches to dementia prevention, which embed preventive measures in local contexts, have been proposed (Walsh et al. 2022; Leibing 2021). In contrast, primary prevention apps seem to reinforce an individualistic perspective, emphasizing personal responsibility *and* equating healthy lifestyles with screen-based, standardized recall exercises.

## Ethical issues of secondary prevention apps

One significant benefit of early detection is the ability to monitor individuals before symptoms appear, allowing for better condition management and potentially slowing disease progression (Vanderschaeghe et al. 2018). Early detection at a stage when cognitive function remains relatively intact also facilitates future planning, such as creating a care plan or enrolling in long-term care insurance (Smedinga et al. 2018; Lohmeyer et al. 2021). Additionally, it has been estimated to reduce the economic burden of dementia. For instance, an older UK study models that delaying the onset of dementia by five years between 2020 and 2025 would reduce the number of people with dementia in 2030 by 36%, significantly lowering care costs (Lewis et al. 2014). Enabling individuals to live independently for longer periods could also result in substantial healthcare cost savings. Thus, early detection is a cornerstone of secondary dementia prevention. The development of biomarkers in recent years has further improved the ability to predict whether a person is at higher risk of developing dementia, even before symptoms arise (Vanderschaeghe et al. 2018). Biomarkers are already widely used in clinical practice, such as in hospitals and memory clinics across Germany (Schweda et al. 2018; Bartels et al. 2020). However, early detection may also lead to psychological burdens (Schürmann and Trachsel 2023). One contributing factor is the (yet) lack of a cure for dementia, which can make early knowledge distressing. Additionally, early detection often involves uncertainty (Smedinga et al. 2018; Alpinar-Sencan et al. 2020). For example, a diagnosis of MCI cannot predict 100% progression to Alzheimer’s disease (AD). An ethical concern surrounding biomarker-based testing in memory clinics, which frequently recommend such tests, is balancing the right to know against the right not to know (Mattsson et al. 2010; Schicktanz et al. 2024). For some individuals, gaining knowledge about a disease with uncertain onset and no cure may offer little benefit. These individuals may exercise their “right not to know,” which, as Herbst (2021) suggests, does not merely involve rejecting specific information but reflects the freedom not to engage in understanding or acting on that information.

While most biomarker-based testing remains embedded within clinical care, the challenges surrounding testing for dementia and AD risk are amplified in the context of self-testing apps. A key selling point of *BrainTest* is its promise of anonymity and independence from healthcare providers—features that could otherwise assist in interpreting results and offering support. The app appeals to individuals who might avoid discussing memory concerns with their doctor out of fear or embarrassment: “We empower you to overcome your fears; nobody will know you’re taking a brain test” (informational video on the company’s website). While test results are accompanied by an explanatory video, further consultation is unavailable, and no support structures are in place for individuals alerted by the app to a potential risk of developing dementia (Ford et al. 2023). Without professional guidance, users are ultimately left to grapple alone with the results or the recommendation to consult a doctor. Difficulties in contextualizing such information and assessing its relevance to one’s own life history are compounded when considering that many dementia-testing apps lack scientific validation (though BrainTest is based on a well-researched tool). Apps of questionable reliability (Charalambous et al. 2020) can lead to false positives, causing unnecessary distress while even valid tools might lead to users misinterpreting their risk (Ienca et al. 2018; Zeiler et al. 2023).

As self-testing apps for early detection become increasingly widespread, the validity of screening tools used outside controlled settings emerges as a critical concern. Validity must address not only medical accuracy but also the broader unintended consequences of self-testing. Recent research suggests that such apps, rather than alleviating fears about dementia or AD, may intensify the very anxieties they aim to mitigate (Kapeller and de Boer 2024). Fear of test results and a potentially bleak future self is closely linked to the stigma surrounding dementia and AD. This stigma can profoundly affect individuals’ relationships with family, friends, and colleagues, as well as contribute to self-stigmatization (see Dubois et al. 2016).

Additionally, there is a risk of discrimination if third parties, such as insurance companies or employers, gain access to early detection results. Individuals identified as being at risk for AD may face denial of employment opportunities or health insurance coverage (Arias and Karlawish 2014). To ensure that self-tests do not fall into the same category as predictive biomarker tests done by health professionals there is a need to legally and ethically clarify, that self-testing by mHealth apps are not reportable to insurance companies or other third parties in any case, especially if they are covered by public health services. These general concerns underscore therefore the importance of carefully evaluating self-testing tools—not only for their technical efficacy but also for their psychological and social implications, including the stigma associated with both the testing process and the resulting diagnoses. This has yet not been sufficiently done.

## Ethical issues of tertiary prevention apps

One of the most pressing ethical issues surrounding tertiary prevention is the risk of indirectly reinforcing stigma through preventive measures. The term “stigma” refers to the perception of an individual as diminished, different, unclean, or in some way less than fully human (Rewerska-Juśko and Rejdak 2020). Public stigma toward individuals with dementia is shaped by common misconceptions, such as the belief that they lack autonomy entirely, are wholly dependent on caregivers, or face fundamental challenges in communicating with others (Werner and Kim 2021). This stigma can lead to hesitation in seeking healthcare, restricted access to appropriate services, social isolation, and even suicide (Günak et al. 2021). It also impacts families caring for people with dementia (Riley et al. 2014; Van den Bossche and Schoenmakers 2022). Family stigma arises when caregivers face similar prejudices to those directed at the person with dementia or when they internalize societal biases against their loved one. Caregiver self-stigma can result in negative emotions, such as unhappiness and helplessness, and is linked to increased caregiver burden, stress, social withdrawal, and reduced willingness to seek support (Werner et al. 2012; Burgener et al. 2015). This type of stigma diminishes the quality of life not only for the person with dementia but also for their caregiving relatives. Various measures are being implemented to combat stigmatization (Bacsu et al. 2022). However, these efforts may be undermined by the growing (over-)expectation that dementia is entirely preventable. Tertiary prevention, when focused narrowly on standardized pharmacological interventions, risks overlooking the diversity of dementia symptoms and thereby perpetuating stereotyped and stigmatized concepts of AD (see Keuck 2021, on the conceptual foundations of AD in prevention). While primary and secondary prevention often emphasize lifestyle-related risk factors, tertiary prevention—seen as treatment—may similarly adopt an individualized approach that implies individuals are responsible for developing AD. This framing can influence caregivers’ attitudes toward patients, potentially reinforcing blame and stigma within care relationships (Petersen 2024).

Ethical debates surrounding the use of mHealth or assistive technologies in dementia and Alzheimer’s care are as old as the technologies themselves. Tracking devices, care robots, and wearables are among the most frequently discussed tools. While mHealth apps in the tertiary sector are primarily developed for caregivers (Kim et al. 2021; Jagoda et al. 2024; Kelley et al. 2024), some are designed to assist patients directly—helping them remember daily tasks, manage medications, or simply enjoy video games. A significant issue with many existing tertiary prevention apps is their failure to align with the values and needs of end-users (Bateman et al. 2017; Eggink et al. 2021). Users prefer apps that provide personalized exercise, sleep, and diet programs rather than generalized solutions (Zhang et al. 2022). Similarly, individuals with dementia, who often enjoy looking at old photos or listening to familiar music, tend to favor personalized media over generic alternatives (Ryan et al. 2020). The app *SingFit*, developed by Musical Health Technologies, addresses caregivers and facilitates singing sessions with patients, either individually or in groups, without requiring prior knowledge of music or music therapy. Featuring an icon of a broad smiling mouth on an orange background, the app conveys qualities that blend the playful appeal of a toy with the seriousness of a health-promoting tool, much like *Lumosity*. *SingFit* includes a music library curated to match the preferences of specific age groups. It allows participants to sing along without needing to read or memorize lyrics, as the lyrics are spoken aloud just before they need to be sung. This feature enables what the company describes as a “failure-free activity,” allowing older adults to engage actively and feel successful despite cognitive impairments. Following each song, the app encourages facilitators to discuss the music with participants, linking it to biographical memories. In this way, a generalized selection of songs (tailored by age group and ability) becomes a personalized exercise or group experience.

Unlike many mHealth tools, the primary interaction prompted by *SingFit* occurs between facilitators and participants rather than between users and the app itself. This approach fosters human connection rather than isolation. The app has received multiple awards for caregiving, telemedicine, and innovation in senior care. One of its co-founders, a music therapist, emphasizes the company’s vision of using the app not only as a complementary treatment for cognitive impairments (see Bleibel et al. 2023; for the medical value of music therapy), but as an alternative to conventional tertiary prevention measures: “Our goal is that 10 years from now, when a child gets diagnosed with ADHD, prescribing a *SingFit* singing intervention to improve focus is as common or more common than prescribing pharmaceuticals.”^10^ The CEO of *SingFit* has similarly expressed the ambition to reintegrate music as a healthcare tool, equating its importance to that of good nutrition.^11^ The image of dementia conveyed by *SingFit* is not one of cognitively impaired individuals confined to passivity but rather of active participants engaging in collective musical activities, stimulated not only cognitively both also vocally and physically. This example demonstrates that the use of an app need not confine individuals to screens, nor does it inherently lead to isolation, responsibilization, or reductionist portrayals of dementia. The app does not replace doctor-patient or caregiver-patient relationships, but builds on it and promises to add a musical dimension to it.

## Data protection and informed consent

Effective customization of mHealth apps for dementia prevention requires the collection of large amounts of personal data. However this raises significant ethical concerns around data protection for all the apps discussed so far. Health data can be used to predict individual needs, personalize support, integrate technology into daily life, and identify people at risk (Robillard et al. 2018; Astell et al. 2019; Hafdi et al. 2021). Ethical issues surrounding the acquisition of such data in the context of dementia prevention technologies include concerns about privacy and the need for informed consent (Piendel et al. 2023).

Legal frameworks and regulations for mHealth apps vary internationally (for the German legal definition of mHealth apps and date protection see Reitebuch 2023). In the U.S., for example, many mHealth apps, despite the dynamic landscape of health data regulations, remain outside the scope of federal privacy laws and are not subject to state or federal regulation (Rosenfeld et al. 2017). As a result, privacy protection for these apps is primarily governed by the companies’ privacy policies. However, many of these policies fail to specify whether user data is sold, potentially prioritizing developers’ rights to exchange data with business partners and third parties, while undermining users’ interests in fair data sharing (Rosenfeld et al. 2017). In Germany standards for digital health applications (DiGA) are strict (Jorzig and Kellermeier 2021), and the Federal Institute for Drugs and Medical Devices (BfArM) is currently developing a certificate for producers of DiGAs to prove their conformity with data protection regulations (Wagner and Harle 2023). But medical devices that are not registered as DiGAs and are classified in the low risk category (such as *memodio*) do not underly specific requirements regarding health data protection (Kirsten et al. 2022; Spranger 2023). Additionally, many apps rely on selling data to advertisers, app developers, or other third parties as part of their business model (Iwaya et al. 2023). All four apps analyzed in this paper address data sharing in their terms of service: two apps (*memodio* and *Lumosity*) explicitly state they will not sell collected data for marketing purposes, while two apps (*BrainTest* and *SingFit*) claim they will not do so without user consent. All four apps share data with third parties to improve app performance. From the perspectives of self-determination and empowerment, it can be argued that users should have the ability to decide the extent to which their data is used. Achieving this requires proper informed consent. There are various challenges regarding informed consent for the use of dementia prevention apps in primary, secondary, and tertiary prevention. In the case of primary prevention apps used by individuals without cognitive impairment, the traditional model of informed consent is difficult to apply (Fisher et al. 2018; Mikkelsen et al. 2019). Informed consent in medical care settings typically involves providing individuals with oral and written information about the benefits, risks, and alternatives of a particular treatment or research project, which they then understand and agree to (Beauchamp and Childress 2019). However, applying this model to mHealth apps for dementia prevention presents several issues. First, obtaining study-by-study consent is impractical, as large amounts of digitized data are often stored for long periods and can be used for multiple studies (Fisher et al. 2018). This complexity creates uncertainty about what information must be disclosed during the consent process regarding potential future uses of the data. Additionally, obtaining study-specific consent can be time-consuming, expensive, varies from app to app, and is often impossible to follow up on later (Cumyn et al. 2019). To address this, a broad consent model has been proposed and partially implemented, which acquires consent for the use of data in multiple unspecified present and future studies (Thorogood et al. 2018). However, this model has faced criticism, as individuals may give consent for future data use without fully understanding what they are agreeing to (Sheehan 2011).

A common concern regarding informed consent in primary, secondary, and tertiary prevention apps is that users often find the privacy policy difficult to understand (Kaplan and Ranchordás 2019). Privacy policies are typically written in complex and regulatory language, which users may not fully comprehend, making it difficult for them to provide truly informed consent. For users of secondary prevention apps, such as those diagnosed with MCI who may already have impaired cognitive function, even a clearly written privacy policy might be challenging to understand. Ethically, these individuals should be provided with additional support to ensure they understand what they are consenting to Prusaczyk et al. (2017). Consumer control mechanisms could ensure the conditions necessary for informed consent in mHealth apps for people with MCI or early stage dementia. In the case of tertiary prevention apps (we have not looked more closely into mHealth apps for patients), it may be impossible to obtain informed consent from individuals with later stages of dementia. Despite decision support tools, there are situations where individuals with dementia are considered incapable of consenting to data sharing (Thorogood et al. 2018). In these cases, consent is typically obtained from a legally authorized representative (LAR). However, this approach is not viable for individuals who do not have an LAR. To address this issue, one proposed solution is for individuals to express their consent to share data in writing before losing decision-making capacity (Karlawish et al. 2009). However, concerns have been raised about whether these pre-established preferences hold once a person loses decision-making capacity, and whether the individual’s identity remains consistent (Jongsma and van de Vathorst 2015). If these challenges are not addressed, the development of tertiary prevention apps for dementia may be hindered.

The options an app provides for offboarding and unsubscribing can serve as indicators of the company’s actual privacy practices. *Memodio* offers users the option to contact them via email to request the deletion of all stored data. (During our walkthrough, the company responds within 48 h, confirming the data deletion.) *Lumosity* deletes all user-related data (including email addresses) when an account is deleted, which can be done with a single click on the company’s website. *SingFit* informs users that terminating their account will result in the deletion of all personal information within 7 days and provides instructions on how to cancel a subscription. In contrast, *BrainTest* does not allow users to close their accounts independently; instead, they must contact the support team separately for cancellation. Additionally, *BrainTest’s* terms of service, privacy policy, and FAQs do not mention what happens to personal data after unsubscribing.

## Conclusions

This paper reexamines bioethical discussions surrounding dementia prevention in the context of mHealth apps. It demonstrates that ethical issue such as the familiar debate on responsibilization of individuals and subsequently blaming individuals for failing to maintain their own mental health, questions of health data protection and informed consent and popular, stigmatized perceptions of dementia get enhanced in the light of increasing recommendation and use of mHealth apps. The in-depth, qualitative analysis of four exemplary apps for dementia prevention shows also the variety of apps, which relate to the ethical issues in question in different ways, since they offer different user-app interactions, privacy policies and imply varied images of dementia and cognitive impairments. Both the affirmative and the critical discourse on mHealth apps would benefit from a close and systematic look on what these apps actually do.

We believe that not only research on effectiveness and benefit evidence of mHealth apps in general, but particularly for dementia prevention needs to be strengthened, and accompanied by nuanced ethical, empirically sound exploration of apps. We support suggestions that primary, secondary and tertiary prevention end-users should be integrated into app development at the earliest possible stage.

The impact of apps must be assessed against the background of the bigger picture of dementia prevention strategies. It needs to be recognized how harmful the claim that dementia can be prevented by individual action (and app use), can be. The overemphasis on individual responsibility for dementia prevention reinforces the stigma of dementia, exacerbates existing health inequalities, and rolls back collective efforts to address structural and systemic inequalities. However, it is not entirely a bad thing for an individual to take responsibility for their personal health. If no individuals take action to lower their dementia risk, dementia prevention as a whole will go nowhere. We consider the issue of who is responsible for dementia prevention not a zero-sum game, but a question of mutually complementary responsibilities. Both individual and societal frameworks are needed to promote dementia prevention. We argue that the responsibility of state and society is to reduce socio-economic inequalities in primary prevention, govern and regulate secondary prevention and provide means to ensure stigma-free tertiary prevention. Mere promotion of mHealth apps does not fulfill this responsibility.

## Footnotes

1 Since we focus on prevention apps and not on apps for treatment, we choose to analyze *memodio*.

2 https://www.handelsblatt.com/technik/medizin/inside-digital-health/start-up-mindahead-rox-health-ausgruendung-entwickelt-demenz-app/29743728.html (accessed June 9, 2024).

3 https://www.bfarm.de/EN/Medical-devices/Tasks/DiGA-and-DiPA/Digital-Health-Applications/Interesting-facts/_node.html (accessed June 9, 2024).

4 Press material, see https://memodio-app.com/ (accessed June 10, 2024). The app costs EUR 250 for 6 months.

5 See https://www.lumosity.com/en/ (accessed June 10, 2024). Non-paying users have restrictions on game use and features. Various pricing plans are available, and the annual fee as of January 2024 is EUR 59.95.

6 https://www.ftc.gov/news-events/news/press-releases/2016/01/lumosity-pay-2-million-settle-ftc-deceptive-advertising-charges-its-brain-training-program (accessed June 11, 2024).

7 See https://braintest.com/ (accessed June 10, 2024). After a free trial, the semiannual plan costs USD 24.99 (a test is recommended every 6 months). The subscription automatically renews.

8 See https://www.singfit.com/ (accessed June 11, 2024). SingFit offers different versions. SingFit STUDIO Caregiver costs USD 11.99 per month, SingFit STUDIO Prof USD 14.99 per month. The subscription automatically renews.

9 See https://www.singfit.com/ (accessed June 11, 2024).

10 https://www.forbes.com/sites/georgehoward/2015/10/01/music-heals-singfit-enables-the-mass-distribution-of-music-as-medicine-to-treat-alzheimers/ (accessed July 1, 2024).

11 https://www.docwirenews.com/post/rachel-francine-ceo-of-singfit-a-music-therapy-platform-that-can-benefit-millions?uid=%3Fuid%3D?uid= (accessed July 1, 2024).
